# Editorial: Metabolism of anti-cancer drugs: Interplay involving pharmacology and pharmacokinetics

**DOI:** 10.3389/fonc.2022.972718

**Published:** 2022-07-29

**Authors:** Tashinga E. Bapiro, Rose Hayeshi, Collet Dandara

**Affiliations:** ^1^ Drug Metabolism & Pharmacokinetics (DMPK), Oncology R&D, AstraZeneca, Cambridge, United Kingdom; ^2^ Department of Science and Innovation/North-West University (DSI/NWU) Preclinical Drug Development Platform, Faculty of Health Sciences, North-West University, Potchefstroom, South Africa; ^3^ Division of Human Genetics, Department of Pathology & Institute of Infectious Disease and Molecular Medicine, University of Cape Town, Cape Town, South Africa; ^4^ Platform for Pharmacogenomics Research and Translation Extramural Unit, South African Medical Research Council, Cape Town, South Africa

**Keywords:** pharmacokinetics, anticancer drugs, pharmacodynamics, drug metabolism, drug-drug interactions, cytochrome P450

One of the hallmarks of cancer is altered energy metabolism as a result of dysregulated pathways (1) which raises the intriguing question regarding the possible effects of dysregulated pathways linked to drug metabolising enzymes and transporters. This Research Topic, therefore, sought to shed more light on the possible impact cancer-associated changes in drug metabolism could have on the pharmacokinetics (PK) and pharmacodynamics (PD) of anti-cancer drugs (2) including any interplay between PK and PD. Studies in patients demonstrating cancer-altered expression of enzymes/proteins and/or associated effects on PK/PD were therefore, of interest in this Research Topic.

The first article focused on rituximab whose PK properties while exhibiting a relationship with clinical response are not well understood particularly regarding the determinants which could include expression levels of CD20 (3 and references therein). In a prospective study running from 2016 – 2021 and involving 51 patients with six B-cell lymphoma subtypes, Liu et al. in this Research Topic, measured rituximab concentrations in plasma. Consistent with previous studies, they showed a direct relationship between rituximab concentrations and response – patients who achieved a complete response had higher trough concentrations than those who did not. In addition, the trough concentrations seemed to be dependent on the lymphoma subtype and negatively correlated with baseline CD19^+^ lymphocyte counts. Current and future efforts to optimise rituximab dosing regimens should benefit from this study with lymphoma subtype and CD19^+^ lymphocyte counts constituting key factors for consideration as suggested by the authors.

The second article in this Research Topic demonstrates the importance of metabolic capacity on the PD of docetaxel. The article was included even though the altered metabolic capacity was genetic rather than cancer-driven – we think this highlights the potential significance of any cancer-associated changes in metabolic capacity. Docetaxel, a commonly used drug for treatment of solid tumors exhibits potentially fatal toxicities associated with increased exposure (4). Cytochrome P450 3A4 mediates the clearance of docetaxel and its activity is, therefore, a key determinant of exposure to the drug. Powell et al. in this Research Topic reported potentially fatal docetaxel toxicity in two patients homozygous for the CYP3A4*22 allele associated with reduced metabolic capacity (5). The absence of docetaxel plasma concentration measurements was an obvious limitation acknowledged by the authors and should be considered in any future studies.

While strategies for predicting human PK of small molecules are well advanced, this is not the case for the not-so-common modalities such as antisense oligonucleotides (ASOs). ASOs are single stranded multi-nucleotides that can be used to treat diseases *via* modulation of protein expression. Our current knowledge of the metabolism of ASOs is limited with only about ten of them FDA-approved at this point in time (6). It is against this background that the Research Topic includes work from Bai et al. who studied the PK of an antisense oligonucleotide in monkeys and used a physiologically based pharmacokinetic model (PBPK) to predict human PK. In this study, the authors were able to validate their PBPK modelling strategy in the monkey and extrapolated this to human. The study should be a useful reference for others working with similar modalities and it will be interesting to see how well this performs when human clinical data from cancer patients becomes available.

In the last article Gabel et al. addressed the classical assessment of the potential for clinically significant pharmacokinetic drug-drug interactions involving two important drugs in oncology, morphine and tamoxifen. It is known that morphine is metabolised *via* glucuronidation and that tamoxifen is a weak inhibitor of glucuronidation (IC_50_ values ~ 100 µM, 7). However, in this Research Topic Gabel et al., looked at the effect of morphine on tamoxifen metabolism and demonstrated a significant increase in glucuronides of both 4-hydroxy-tamoxifen and endoxifen by morphine *in vivo* in mice which they did not expect as data from their *in vitro* work in mouse microsomes suggested inhibition of glucuronidation by morphine as the mechanism of interaction. While the study, as the authors suggest, highlights the possible pitfalls in extrapolation of *in vitro* data to the *in vivo* situation, the differences in the concentrations used is an important point to note. The much higher concentration of morphine (500 µM) used *in vitro* compared to 1 µM achieved *in vivo* in the mice and the known fact that tamoxifen modifies its own metabolism should be useful points to consider in any efforts to better understand the mechanism of interaction including the discordance between the *in vitro* and *in vivo* data. As the effects of tamoxifen on its own metabolism are thought to involve the estrogen receptor and various transcription factors, future studies could investigate whether these interactions would be modified in cancer preclinical models or patients.

The articles in this Research Topic address aspects of metabolism of anti-cancer drugs in a small way that highlights the need for more studies to shed light on the possible fascinating effects of cancer-associated changes in drug metabolism and how this knowledge could be harnessed to come up with better treatment regimens.

## Author contributions

All authors contributed to the conception and design of the Research Topic. All authors wrote sections of the editorial. All authors contributed to manuscript revision, read, and approved the submitted version.

## Conflict of interest

Author TB was employed by AstraZeneca.

The remaining authors declare that the research was conducted in the absence of any commercial or financial relationships that could be construed as a potential conflict of interest.

## Publisher’s note

All claims expressed in this article are solely those of the authors and do not necessarily represent those of their affiliated organizations, or those of the publisher, the editors and the reviewers. Any product that may be evaluated in this article, or claim that may be made by its manufacturer, is not guaranteed or endorsed by the publisher.

## References

[1] Hanahan D Weinberg RA. Hallmarks of cancer: The next generation. Cell (2011) 144:646–74. doi: 10.1016/j.cell.2011.02.013 21376230

[2] Grasso C Jansen G Giovannetti E. Drug resistance in pancreatic cancer: impact of altered energy metabolism. Crit Rev Oncol/Hematol (2017) 114:139–52. doi: 10.1016/j.critrevonc.2017.03.026 28477742

[3] Cartron G Blasco H Paintaud G Watier H Le Guellec C. Pharmacokinetics of rituximab and its clinical use: Thought for the best use? Crit Rev Oncol/Hematol (2007) 62:43–52. doi: 10.1016/j.critrevonc.2006.09.004 17287129

[4] Kenmotsu H Tanigawara Y. Pharmacokinetics, dynamics and toxicity of docetaxel: Why the Japanese dose differs from the Western dose. Cancer Sci (2015) 106(5):497–504. doi: 10.1111/cas.12647 25728850 PMC4452149

[5] Mulder TAM van Eerden RAG de With M Elens L Hesselink DA Matic M. *CYP3A4^∗^22* genotyping in clinical practice: Ready for implementation? Front Genet (2021) 12:711943. doi: 10.3389/fgene.2021.711943 34306041 PMC8296839

[6] Migliorati JM Liu S Liu A Gogate A Nair S Bahal R. Drug metabolism and disposition. (2022). doi: 10.1124/dmd.121.000417 PMC11022858 35221287

[7] Hara Y Nakajima M Miyamoto K Yokoi T. Morphine glucuronosyltransferase activity in human liver microsomes is inhibited by a variety of drugs that are Co-administered with morphine. Drug Metab Pharmacokinet (2007) 22(2):103–12. doi: 10.2133/dmpk.22.103 17495417

